# Editorial: Intervertebral disc degeneration and osteoarthritis: mechanisms of disease and functional repair

**DOI:** 10.3389/fbioe.2023.1252703

**Published:** 2023-07-26

**Authors:** Graciosa Q. Teixeira, Jana Riegger, Raquel M. Gonçalves, Makarand V. Risbud

**Affiliations:** ^1^ Institute of Orthopaedic Research and Biomechanics, Trauma Research Centre, Ulm University, Ulm, Germany; ^2^ Department of Orthopedics, Division for Biochemistry of Joint and Connective Tissue Diseases, Ulm University, Ulm, Germany; ^3^ Instituto de Investigação e Inovação em Saúde, Universidade do Porto, Porto, Portugal; ^4^ Instituto de Engenharia Biomédica, Universidade do Porto, Porto, Portugal; ^5^ Instituto de Ciências Biomédicas Abel Salazar, Universidade do Porto, Porto, Portugal; ^6^ Department of Orthopaedic Surgery, Sidney Kimmel Medical College, Thomas Jefferson University, Philadelphia, PA, United States; ^7^ Graduate Program in Cell Biology and Regenerative Medicine, College of Life Sciences, Thomas Jefferson University, Philadelphia, PA, United States

**Keywords:** intervertebral disc (IVD), cartilage, degeneration, biomechanics, inflammation, regeneration, tissue engineering

Intervertebral disc (IVD) degeneration and osteoarthritis (OA) are the leading musculoskeletal causes of disability worldwide, with a considerable loss of productivity and high healthcare costs. Both diseases are associated with severe pain, affecting about 90% of the over 50 years old (IVD) and more than 50% of the over 65 years old (OA) population. Current treatments, varying from physiotherapy to invasive surgery, carry high costs. These approaches may alleviate pain and improve mobility in the short run, but do not target the underlying causes of tissue degeneration. IVD, articular cartilage, and meniscus are tissues that share some similarities in cellular metabolism and matrix homeostasis in health and disease. Particularly during pathogenesis, there is a strong interplay between tissue degradation, inflammation, and overall immune response, which has not been fully understood so far.

It is essential to identify pathophysiologic key molecules and pathways that could be targeted by means of molecular medicine, pharmacologic, and tissue engineering strategies to promote cartilaginous tissue repair/regeneration, mainly by modulating the immune response. It should be considered that similar approaches might be effective for IVD, articular cartilage, and meniscus. Especially in the case of young, active patients, regenerative strategies including administration of bioactive molecules and cell transplantation alone or in combination with scaffolds, have been investigated for IVD regeneration and articular joint repair over the past two decades, showing promising results *in vivo* and in clinical trials. Nevertheless, for the development of innovative, off-the-shelf, and long-term effective regenerative strategies, it is important to understand the molecular mechanisms underlying the disease and, in particular, the interplay between matrix breakdown and the local and systemic immune response. For instance, the affected IVD or joint presents a hostile inflammatory environment challenging for the long-term effect of homing tissue cells or engraftment of transplanted cells. Therefore, there is an urgent need for alternative approaches to modulate inflammation and matrix degeneration, in order to stimulate tissue repair/regeneration.

This Research Topic includes a Research Topic of six articles that address a broad range of relevant questions including a work investigating the delamination of annulus fibrosus using bovine model (Briar et al.), a study on the role of aggrecan in the maintenance of the physiologic stiffness of the IVD (Empere et al.), and the development of a human 3D nucleus pulposus (NP) microtissue (µT) model to evaluate the potential of pre-conditioned nasal chondrocytes for the repair of degenerated IVDs (Kasamkattil et al.). In addition, Habib et al. investigated the effect of intradiscal delivery of a matrix-modifying enzyme to the cartilage endplate. These publications contribute to a better understanding of the physiology and biomechanical characteristics of the different IVD compartments and outline new potential therapeutic strategies to improve the biomechanical behavior of the degenerated IVD. In addition, this Research Topic comprises two publications focused on cartilage and OA. The work from Riegger et al. investigated the effect of simvastatin and fluvastatin to attenuate cell death and catabolism in human cartilage after *ex vivo* trauma. Lastly, an opinion article on the usage of low-level laser therapy in patients with knee joint OA summarized the relevant literature on the effectiveness, as well as implications of this therapeutic approach (Khumaidi et al.).

Briar et al. investigated the influence of delamination rate on the adhesive properties of the layer-adjoining interlamellar matrix of annulus fibrosus from bovine tail IVDs at different rates of separation. The annulus fibrosus stretches under tension and, therefore, this is a relevant work to better understand the mechanical behavior of this under-investigated IVD compartment (in comparison to the NP), as the spine experiences various loading velocities during activities of daily living. Overall, no differences in lamellar adhesion strength or stiffness were observed across all loading rates. However, lamellar adhesion strength variability was observed to increase with an increasing rate of delamination, possibly highlighting a viscoelastic response to this structure. According to the authors, such mechanical behavior may permit the development of microtrauma at lower overall levels of force, and thereby present an injury scenario in which the disc becomes more susceptible to delamination (Briar et al.).

Empere et al. provided a deeper understanding of aggrecan (ACAN) within the IVD compartments by taking advantage of genetically engineered mouse models. By generating an ACAN insertion mutant mice (AcaniE5/iE5) the authors reported compressed vertebral bodies with accelerated mineralization in ACAN mutants compared to wild type controls. Furthermore, collapsed extracellular matrix with negligible sulfated glycosaminoglycan content accompanied by a high cellular density was observed in AcaniE5/iE5 mice, without impairing collagen type II deposition. Severe alterations in cartilage endplates (CEP), NP structure, and growth plate were observed, from the macroscopic to the nanoscale level. Taken together, the team demonstrated the importance of aggrecan to the maintenance of the proper stiffness of IVD and vertebral tissues.

In addition, Kasamkattil et al. developed an advanced 3D NPµT model that could be used to test the performance of nasal chondrocytes (NC) suspension or spheroids (NCS) after pre-conditioning with drugs known to exert anti-inflammatory or anabolic activities. Degenerative NPµT contained lower concentration of glycosaminoglycans and collagens and released higher levels of interleukin-8 (IL-8) compared to the healthy NPµT. Interleukin-1 receptor antagonist (IL-1Ra) pre-conditioning inhibited the expression of inflammatory/catabolic mediators and promoted glycosaminoglycan accumulation in NC/NCS in the degenerative IVD microenvironment. The degenerative NPµT model is suitable to study the responses of therapeutic cells to microenvironment mimicking early-stage degenerative disc disease. NC in spheroidal organization exhibited superior regenerative performance compared to NC cell suspension. IL-1Ra pre-conditioning of NCS could further improve the NC ability to counteract inflammation/catabolism and support new matrix production within the harsh degenerative microenvironment in disc disease.

CEP nutrient transport, a key factor in the etiology of intervertebral disc degeneration, was also addressed in the present Research Topic. Habib et al. tested whether intradiscal delivery of a matrix-modifying enzyme to the CEP improves solute transport into whole human and bovine discs. In fact, the enzymatic treatment of the CEP dramatically enhanced small solute transport in both tissue models, without affecting disc biomechanical behavior, demonstrating a potential benefit for matrix modification of the CEP to promote the transport of small solutes into whole intact IVDs.

In their study on the therapeutic effects of Fluvastatin and Simvastatin after *ex vivo* cartilage trauma, Riegger et al. observed cell and chondroprotective effects for both statins. However, the potent anti-catabolic and antioxidative properties of the drugs also compromised chondrogenic re-differentiation of human chondrocytes, when permanently applied, as demonstrated in a 3D pellet culture model. Although chondrogenic re-differentiation was recovered and even increased after short-term treatment using low concentrations of Simvastatin, this concentration was less effective in cell protection after cartilage trauma. The authors concluded that statin therapy does not directly contribute to cartilage regeneration, but might be a suitable approach to prevent injury-induced cartilage degeneration and thus reduce the risk of posttraumatic OA.

The opinion article from Khumaidi et al. argues that low level laser therapy (LLLT), which is the electronic level absorption of laser light without the production of heat in the visible to near infrared spectral spectrum (390–1100 nm), can be used as a non-invasive treatment modality for knee OA patient. Based on literature findings, the authors defend that in combination with physical exercises, LLLT may result in reduced inflammation, improvement in Western Ontario and McMaster Universities Osteoarthritis Index (WOMAC) scores, pain, range of motion, and functional status in knee OA patients via photobiomodulation. However, Khumaidi et al. call for rigorous and standardized research to overcome current variability in laser application research methodology and the lack of laser type data, dose range research, and wavelength selection.

The articles in this Research Topic elucidate the behavior of IVD structures and mechanisms involved in IVDD and post-traumatic OA, and/or propose novel therapeutic approaches for the cartilaginous structures. We hope this contributes to a better understanding of the degeneration-associated processes and that the proposed therapeutic approaches may have the potential to improve patient care in the future.

